# Relationship between disgust and orthorexia nervosa and psychometric properties of the Italian Dusseldorf orthorexia scale in a general population sample

**DOI:** 10.1186/s40337-023-00899-5

**Published:** 2023-10-03

**Authors:** Matteo Aloi, Martina Moniaci, Marianna Rania, Elvira Anna Carbone, Gabriella Martino, Cristina Segura-Garcia, Marco Tullio Liuzza

**Affiliations:** 1 Department of Clinical and Experimental Medicine, University of Messina, Messina, Italy; 2 Department of Health Sciences, University “Magna Graecia” of Catanzaro, Catanzaro, Italy; 3 Outpatient Unit for Clinical Research and Treatment of Eating Disorders, University Hospital “Mater Domini”, Catanzaro, Italy; 4 Department of Medical and Surgical Sciences, University “Magna Graecia” of Catanzaro, Viale Europa, 88100 Catanzaro, Italy

**Keywords:** Orthorexia nervosa, Measurement invariance, Error covariance, Disgust sensitivity, Nomological validity

## Abstract

**Background:**

It remains unclear among clinicians and researchers whether orthorexia nervosa (ON) is a part of the obsessive–compulsive disorder spectrum or eating disorders. Disgust seems to be a shared psychopathological factor in these clinical presentations, indicating a potentially crucial role in ON. On the other hand, numerous psychometric tools have been developed to evaluate ON. The Dusseldorf Orthorexia Scale (DOS) was recently validated in an Italian sample. However, the study's primary limitation was that the scale was only administered to undergraduate university students. This study aimed to investigate the psychometric properties (including factorial structure, reliability, and measurement invariance conditional on sex) of the Italian version of the DOS (I-DOS) on a sample from the general population. Additionally, the study sought to determine the nomological validity of the I-DOS by examining its relationship with disgust sensitivity.

**Methods:**

A sample of 521 participants took part in this study and completed a battery that assessed ON and disgust sensitivity. To assess the I-DOS structure, reliability, and measurement invariance we respectively conducted confirmatory factor analysis (CFA), computed McDonalds’s omega, and performed hierarchical series of multigroup CFAs. Then, we tested the relationship between ON and disgust sensitivity.

**Results:**

CFA confirmed the unifactorial model of I-DOS and it respected the configural, metric, and strict invariance while a partial scalar invariance was achieved. It also showed good reliability with an omega of 0.87. In addition, we found a positive relationship between ON and disgust sensitivity, thus confirming the nomological validity of I-DOS.

**Conclusions:**

Our findings suggest that the Italian version of the Dusseldorf orthorexia scale (I-DOS) exhibits strong psychometric properties and can be an effective instrument for assessing ON in a general population sample. Notably, the most significant and innovative outcome was the positive correlation between ON and disgust sensitivity. As disgust has been linked to other clinical presentations, this preliminary result could serve as a foundation for future research exploring this phenomenon in greater detail.

**Supplementary Information:**

The online version contains supplementary material available at 10.1186/s40337-023-00899-5.

## Abstract (plain language summary)

This study sought to investigate the psychometric properties of the Dusseldorf Orthorexia Scale (DOS) in an Italian sample from the general population. Furthermore, as disgust seems to be a shared psychopathological factor in orthorexia nervosa (ON), obsessive-compulsive disorder, and eating disorders, we explored the nomological validity of I-DOS testing its relationship with disgust. The results confirmed that I-DOS has sound psychometric properties and, especially, a positive relationship between ON and disgust sensitivity. Therefore, future studies should focus on targeting disgust sensitivity as the focal point in ON.

**Supplementary Information:**

The online version contains supplementary material available at 10.1186/s40337-023-00899-5.

## Background

In 1997, Dr. Steven Bratman observed an obsession with "correct" eating among his patients, which led him to coin the term orthorexia (ON) from the Greek words ὀρθός (right) and ὄρεξις (appetite) [1]. Over the course of the past two decades, several clinicians and researchers have made efforts to develop clear and precise clinical criteria for identifying ON. Despite being a widely studied phenomenon, Orthorexia Nervosa (ON) is not still currently recognized as a distinct mental disorder in the most recent editions of the Diagnostic and Statistical Manual of Mental Disorders (DSM-5-TR) or the International Classification of Diseases (ICD-11). As a result, there is an ongoing debate among experts as to whether ON should be considered a separate mental disorder.

A group of forty-seven experts from fourteen countries across four continents, representing various disciplines, collaborated to sign a consensus document on the definition and diagnostic criteria of ON in the most recent study. In the paper, twenty-seven statements met the consensus threshold and were included in the proposed diagnostic criteria for ON (Criterion A: definition, clinical aspects, and duration; Criterion B: consequences; Criterion C: onset of ON; Criterion D: exclusion criteria; other characteristics associated or possibly risk factors; differential diagnosis with other psychiatric diseases) [2].

Nonetheless, clinicians and researchers have not clarified whether ON pertains to eating disorders (EDs) [3, 4] or the obsessive–compulsive disorder (OCD) spectrum [5, 6]. OCD, especially contamination-related OCD, is characterized by a heightened experience of disgust [7, 8]. Further, disgust was presented as a transdiagnostic feature across EDs [9] from Anorexia Nervosa [10] to Binge eating disorder [11]. Disgust, especially body odor disgust sensitivity [12], is a primary emotion that is supposed to be evolved as a pathogen avoidance mechanism [13], and the obsession with healthy eating might be related to the overactivation of a pathogen avoidance mechanism. This argument could be framed within the background of the behavioral immune system framework (BIS; [14]). The BIS is a set of psychological mechanisms that may have evolved to recognize pathological threat signals thereby activating appropriate affective and cognitive responses and eliciting associated avoidance behaviors [15]. It is not surprising that disgust plays a central role in the BIS. In fact, disgust is an important universal emotion and is considered a defense mechanism to protect the body from contamination by harmful substances [16]. Surprisingly, there are no studies that have investigated the potential relationship between ON and disgust.

In recent years, alongside efforts to establish clear definitions and diagnostic criteria for ON, a variety of psychometric instruments have been developed to measure ON. A recent review has investigated the prevalence of ON as measured by several diagnostic tools for ON [17], namely the ORTO-15 by Donini and colleagues [18], the Eating Habits Questionnaire (EHQ; [19]), the Dusseldorf Orthorexia Scale (DOS; [20]), the Barcelona Orthorexia Scale (BOS; [21]), the Teruel Orthorexia Scale (TOS; [22]) and the Orthorexia Nervosa Inventory (ONI; [23]). None of the tools has been identified as the "gold standard" that is the most suitable tool for the assessment of ON, even if some of them are more promising than others. Many instruments have been criticized for poor validity (i.e. low internal consistency) and failure in other psychometric domains (i.e. inadequate fit model of the factorial structure), particularly the ORTO-15 [24]. The DOS has been validated in different languages and it showed good reliability, criterion validity, and factor structure [25–27]. Further, DOS was recently validated in an Italian sample; however, the main limitation of the study was that the scale was only administered to undergraduate university students [28].

Finally, ON symptomatology has been investigated in terms of its relationship with body mass index (BMI), with results being inconsistent. In fact, some studies have not reported an association between these two constructs [28–30], while others have reported it [31, 32]. The association between orthorexia behaviors and lower BMI may be due to individuals with ON restricting their food choices to "healthy" foods, leading to a lower calorie intake and potential weight loss. However, it is worth noting that ON can occur across a wide range of body weights, and BMI alone does not reflect the full extent of the disorder or an individual's overall health. ON is primarily defined by an unhealthy fixation on healthy eating, rather than specific body weight or composition. Therefore, this association needs further study.

Based on the above, the aim of the present study was twofold: (1) to further explore the psychometric properties (factorial structure, reliability, and measurement invariance conditional on sex) of the I-DOS on a more diverse sample from the general population with a wider age range than the validation study of Cerolini and colleagues [28]; (2) to provide further evidence in support of the nomological validity of I-DOS [33] by testing its theoretically relevant relationship with disgust sensitivity, especially body odor disgust sensitivity.

We expected to find an adequate model fit as measured by the CFA, a good internal consistency (ω_t_ ≥ 0.70), and a positive correlation between ON and disgust sensitivity.

## Methods

### Participants

A convenience sample of five-hundred-twenty-seven participants was recruited from October 2021 to April 2022 among the Italian general population (Table 1). Due to missing data, six (1.1%) participants were dropped from the analyses, so the final sample comprised 521 partakers (response rate 98.8%) of which 326 (62.6%) were female, and 195 (37.4%) were male. The mean age of the participants was 33.9 ± 14.2 years. The mean score of the BMI, derived from self-reported height and weight, was 23.6 ± 3.8 kg/m^2^. Finally, most participants were normal weight, had completed high school and came from Southern Italy.

**Table 1:** Socio-demographics characteristics of the sample

		Total sample			
		*N* = 521		Skewness	Kurtosis
Age^a^		33.9	(14.2)	.750	−.762
BMI^a^		23.6	(3.8)	3.758	36.250
Sex^b^	Men	195	(37.4)		
	Women	326	(62.6)		
Categorical BMI^b^	Underweight	25	(4.8)		
	Normal weight	330	(63.3)		
	Overweight	129	(24.8)		
	1st degree obesity	31	(6.0)		
	2nd degree obesity	6	(1.2)		
Years of education^a^	Elementary	3	(0.6)		
	Middle school	37	(7.1)		
	High school	305	(58.5)		
	Master	157	(30.1)		
	Ph.D	19	(3.6)		
Residence^b^	Southern Italy	462	(88.7)		
	Central Italy	34	(6.5)		
	Northern Italy	25	(4.8)		

^a^Data are presented as means (SD)

^b^Data are presented as frequencies (%)

### Procedure

After dissemination through main social media platforms such as Facebook, Instagram, and WhatsApp, participants were given a link to an online questionnaire created using "Google Forms" to complete.

Before beginning the questionnaire, participants were informed about the research purpose and assured of their data anonymity. They were also informed that withdrawal from the study would not result in any negative consequences. The researchers provided their contact information to address any concerns. Participants were eligible for inclusion if they met the following criteria: being 18 years of age or older, being a native Italian speaker, and providing informed consent. Once they signed the consent form, participants willingly and without any form of incentive or reimbursement completed the survey. The questionnaire took approximately 15 min to complete (see Additional file 1).

### Measures

#### Demographic information

Demographic information regarding sex, age, BMI, place of residence, and level of education was collected (see Table 1).

#### Düsseldorf orthorexia scale (DOS)

The I-DOS was used to measure orthorexic attitudes and behaviors. Cronbach's α of the Italian version was 0.89, showing good internal consistency [28]. The scale is composed of 10 items on a 4-point Likert scale, ranging from 1 (*It does not correspond to my behavior at all*) to 4 (*It corresponds well to my behavior*). The maximum score is 40; with higher scores indicating more pronounced orthorexic behavior. A score of ≥ 30 is considered indicative of the presence of ON, while a score falling between 25 and 29 (95th percentile) indicates the risk of ON [20, 28].

#### Body odor disgust scale (BODS)

The Body Odor Disgust Scale (BODS) was used to assess individual differences in disgust response to a variety of body odors. The BODS was first validated in English [12] and its Italian psychometric properties were assessed within a multi country study [34] The scale is a self-report questionnaire composed of 12 items and presents participants with a series of descriptions of situations (e.g., “You are standing next to a stranger and notice that the t-shirt they are wearing smells strongly from their sweat.”). Participants are asked to report on a five-point Likert-type item ranging from 1 (*Not disgusting at all*) to 5 (*Extremely disgusting*) the degree to which they found the situation disgusting. The tool can be used both as a one-dimensional scale and as a scale that reflects two factors: sensitivity to body odors coming from internal vs. external sources. Higher scores indicate higher levels of body odor disgust sensitivity. The original scale showed excellent internal consistency with Cronbach's α > 0.9. In the present study, Cronbach's α and McDonald’s ω were both 0.92.

#### Three domains of disgust scale (TDDS)

The Three Domains of Disgust Scale (TDDS) is a tool that measures pathogenic, sexual, and moral disgust. The original version of the scale, developed by Tybur and colleagues [35], demonstrated good psychometric properties and a three-factor structure. The Italian version of the scale, as confirmed by exploratory and confirmatory factor analyses, also showed good internal consistency and construct validity in a study conducted by Poli and colleagues [36]. The scale comprises 21 items, with seven items for each subscale. In this study, only the subscale that examines pathogenic disgust, consisting of 7 items, was used. Participants were asked to rate each item on a 7-point Likert scale, ranging from 0 (*not disgusting at all*) to 6 (*extremely disgusting*). Higher scores in this subscale correspond to higher levels of disgust sensitivity to the pathogenic cues. In the present research, Cronbach's α and McDonald’s ω were both 0.74.

### Statistical analysis

All data were processed using the software RStudio R 3.0.1 [37] and SPSS version 22. The Confirmatory Factor Analysis (CFA) was conducted through the robust weighted least squares—means and variance adjusted (WLSMV) estimator and followed the same procedure used by Cerolini and colleagues [28] to test the best factor structure of the model. Therefore, to obtain acceptable fit indices a model with the residual error covariances of items 6 and 10, and of items 4 and 7 was tested. The following fit indices were reported with the recommended values: the root mean squared error approximation (RMSEA; less than 0.08 indicates good fit), Comparative Fit Index (CFI; greater than 0.90/0.95 indicates acceptable/good fit), Tucker-Lewis Index (TLI; greater than 0.90/0.95 indicates an acceptable/good fit), and the standardized root mean square residual (SRMR; less than 0.05/0.08 indicates a good/acceptable fit) [38].

Subsequently, measurement invariance across sexes was tested using a hierarchical series of multigroup CFAs. The following levels of measurements invariance were examined: configural (i.e., same factor structure between groups), metric (i.e., factor loadings equal between groups), scalar (i.e., the equivalence of item intercepts between groups), and strict (i.e., same factor loadings, intercepts, and residual variances between groups). Measurement invariance is supported if the comparison between the two nested models meets the following criteria: a non-significant *p *value (*p* < 0.05) associated with Δχ^2^, ΔRMSEA < 0.050, ΔCFI < 0.004, and ΔSRMR ≤ 0.01. To check the source of the lack of equivalence, R modification indices were also investigated. By the way, when a constraint is untenable, it can be relaxed to obtain partial invariance [39].

Besides, we computed McDonald's omega (ώ) [40] to determine the reliability of the I-DOS.

To test for nomological validity, we built a latent variable model with two latent variables: a latent variable for pathogenic disgust reflected by the items of BODS and TDDS pathogenic subscale and a second latent variable for ON reflected by the item of I-DOS. In order to increase reliability, we putted together BODS and TDDS-pathogenic because they are two closely correlated scales (r = 0.42 in this study). Then, we investigated the relationship between these two latent variables taking into account the measurement error that varies across indicators. Further, Pearson's correlation was calculated to test the possible relationship between I-DOS and BMI.

Finally, independent-sample t-tests were performed to compare the difference in mean scores between sexes in the I-DOS. To determine the magnitude of the effect of the difference, Cohen's *d* was calculated, with the following values: *d* < 0.20; 0.21 < *d* < 0.50; 0.51 < *d* < 0.80; 0.81 < *d* < 1; *d* > 1, considered respectively as a negligible, small, medium, large and excellent effect [41].

Statistical significance was set at *p* < 0.05.

## Results

### CFA of I-DOS

**Fig. 1:**
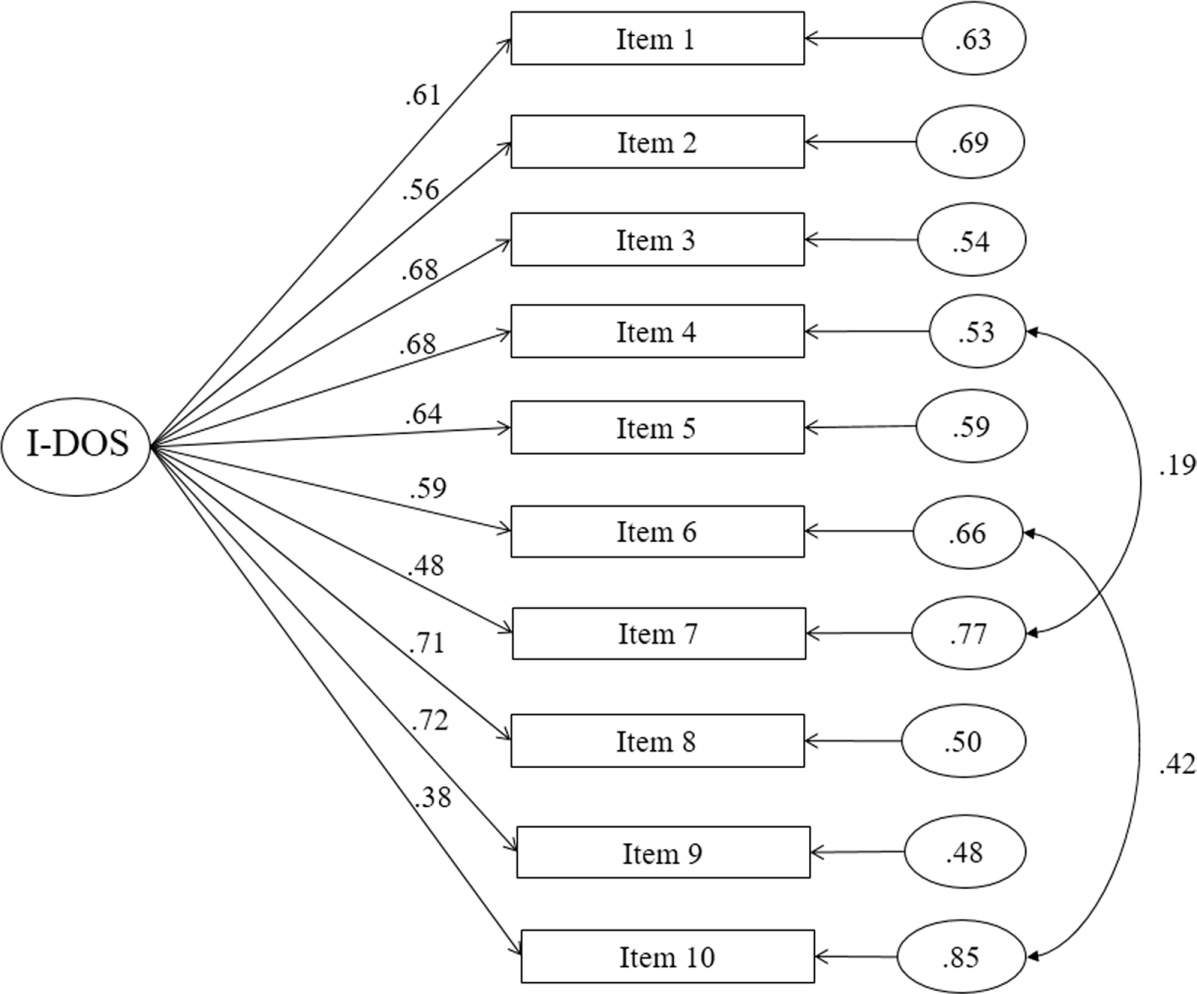
CFA with standardized factor loadings of I-DOS

The results of the CFA indices showed a good fit of the model: CFI = 0.97; TLI = 0.96; RMSEA = 0.08; SRMR = 0.08. The graphical representation of the CFA with standardized factor loadings is shown in Fig. 1.

### Measurement invariance across sexes

Multiple-group CFA was run to examine the measurement invariance across sex. Fit indices for the four models and the differences between the pairs of nested models are displayed in Table 2.

**Table 2:** Fit indices for measurement invariance tests for sex

Robust model fit indices								Model difference						
Model	χ^2^	df	CFI	TLI	RMSEA	SRMR	ΔM	Δ χ^2^	Δdf	p	ΔCFI	ΔTLI	ΔRMSEA	ΔSRMR
M1	153.929	66	0.98	0.97	0.07	0.08								
M2	178.557	76	0.98	0.97	0.07	0.08	M2 VS. M1	2.4985	10	0.99	0.004	0.000	0.000	− 0.006
M3	205.414	85	0.97	0.97	0.07	0.09	M3 VS. M2	21.044	9	0.01	0.004	0.001	− 0.002	− 0.005
M3*	196.877	84	0.97	0.97	0.07	0.08	M3* VS. M2	14.524	8	0.07	0.003	0.000	0.000	− 0.003
M4	222.336	94	0.97	0.97	0.07	0.09	M4 VS. M3*	8.283	10	0.60	0.004	0.000	− 0.001	− 0.007

M1: configural invariance; M2: metric invariance; M3: scalar invariance; M3*: partial scalar invariance; M4: strict invariance

All the Δ χ^2^ were not significant

First, the configural invariance was assessed by estimating both sex groups without equality constraints. The results confirmed the configural invariance of the I-DOS (M1) as indicated by the fit indices.

Then, metric invariance (M2) was achieved by constraining the factor loadings to be the same between male and female groups, and the model fit of this solution was acceptable. Compared with M1, M2 reported that Δχ^2^ was not significant and value changes of CFI (ΔCFI), TLI (ΔTLI), RMSEA (ΔRMSEA), and SRMR (ΔSRMR) were within the recommended threshold for supporting the measurement invariance. These results showed that the metric invariance of the I-DOS held across sexes.

The scalar invariance was assessed by restricting factor loadings and intercepts of items to make them equally between the two sex groups. Results from the scalar invariance model (M3) showed that the model worsened the fit. An inspection of the modification indices revealed a constraint not tenable (threshold of item 1), but a partial scalar invariance model was achieved after it was relaxed. Compared with M2, the values of ΔCFI, ΔTLI, ΔRMSEA, and ΔSRMR were all within the recommended threshold for supporting the measurement invariance and Δχ^2^ was not significant.

Finally, strict invariance was estimated by forcing the factor loadings, intercepts, and residual variances of the items to be the same across the sexes. The strict invariance model (M4) provided acceptable fit indices. Compared with M3, the values of ΔCFI, ΔTLI, ΔRMSEA, and ΔSRMR were all smaller than the recommended cutoff values for rejecting measurement invariance and Δχ^2^ was not significant.

### Reliability and nomological validity

The I-DOS exhibited good reliability as evidenced by the reported value: McDonald's ω coefficient was 0.87, indicating good reliability.

**Fig. 2:**
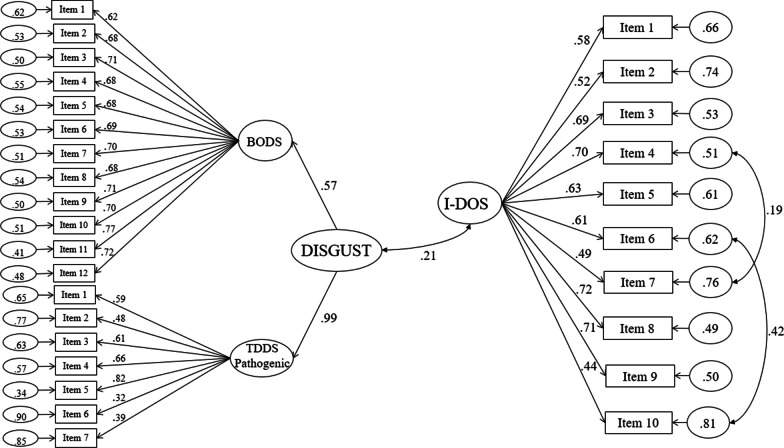
Latent variable model regarding the relationship between disgust sensitivity and orthorexia nervosa

A small positive correlation emerged between the latent variable for ON accounted by the item of I-DOS and the latent variable regarding the construct of pathogenic disgust reflected by the items of BODS and TDDS pathogenic subscale (*r* = 0.21, *p* < 0.001) (Fig. 2). No statistically significant correlation emerged between I-DOS and BMI (*r* = 0.11, *p* = 0.07).

### Comparison of I-DOS score between male and female

No differences emerged in I-DOS total score between males and females (*t* = − 1.81; df = 519; p = 0.07, male = 20.6 ± 6.5, female = 19.5 ± 6.2).

Further, the prevalence of ON in our sample, according to the cut-off of I-DOS ≥ 30, was 7.5% (95% CI 5–9%), while, according to the less restrictive cut-off of a total score between 25 and 29, the prevalence of ON risk was 12.9% (95% CI 10–16%). More in detail, the prevalence of ON in the female was 6.4% (95% CI 3.5–8.5%), while, the prevalence of ON risk was 12.6% (95% CI 9–16%); in the male subgroup, the prevalence of ON and the ON risk was 9.2% (95% CI 5.2–13.2%) and 13.3% (95% CI 8.3–17.7%) respectively.

## Discussion

The present study aimed to test the psychometric properties and measurement invariance of the I-DOS and its relationship with disgust sensitivity in a general population sample.

The CFA indices confirmed a good fit of the unifactorial model of I-DOS. We retested the unifactorial model of Cerolini and colleagues that correlated error covariances of items 4 and 7 and of items 6 and 10. Regarding items 6 (“*If I eat something I consider unhealthy, I feel really bad*”) and 10 (“*I feel upset after eating unhealthy foods*”), a previous study suggested correlating their error covariances because they refer to negative feelings as a consequence of consuming unhealthy food [29]. Further, for items 4 (“I *try to avoid getting invited over to friends for dinner if I know that they do not pay attention to healthy nutrition*”) and 7 (“*I have the feeling of being excluded by my friends and colleagues due to my strict nutrition rule”*), Cerolini and colleagues proposed to correlate them since the common theme of these items seems to be related to the interpersonal and social functioning which are central aspects of ON assessed by the DOS [28].

Regarding reliability, Cerolini and colleagues used Cronbach's alpha while in our study we used McDonald's ώ because it does not have restrictive assumptions like alpha [42, 43]. The omega in our study suggested good internal consistency (ώ = 0.87) and was in line with other DOS validation studies using the omega, such as the Polish [27] and French [44] versions that reported good values as 0.84 and 0.87, respectively.

Concerning the measurement invariance between the sexes, the I-DOS showed configural, metric, and strict invariance while a partial scalar invariance was achieved. Specifically, in the scalar invariance, item 1 (“Eating healthy food is more important to me than indulgence/enjoying the food”) was non-invariant, indicating that males and females perceived this item differently. More in detail, our findings showed that females had a higher intercept value for this item compared to males. This suggests that, when the observed scores are equal, females may have higher levels in the latent trait or that this difference may be due to a response bias.

Interestingly, we did not find an association between I-DOS and BMI in the present research. This finding might indicate that ON is unrelated to weight, as previous studies demonstrated [28–30]. In other words, weight and body image concerns seem closely related to patients with EDs rather than people with ON, who are concerned with food quality rather than quantity. Further, recent research found that ON symptomatology is greater for individuals with a higher BMI [45, 46]. A possible explanation is that ON can develop through the concerted effort of individuals who initially have relatively high body fat to switch to a healthy diet to achieve optimal body weight and improve physical health.

The most novel result of our study was the positive although small relationship between ON and disgust sensitivity. Research has examined the connection between disgust and eating disorders [47] and, although there is not a direct link to eating psychopathology, the presence of anxiety symptoms may help explain its association in these individuals [48]. In the specific case of ON, the disgust towards foods perceived as unhealthy can be a mechanism of perpetuation and maintenance of orthorexic behaviors and attitudes [49]. In fact, an important theory of disgust has hypothesized that disgust is an adaptive response to food refusal, noting the relationship between a physiological correlate of disgust, namely nausea, and the expulsion of inappropriate foods; moreover, disgusting objects that have negative sensory properties such as a bad taste, smell, or texture tend to be rated as unpleasant [50, 51]. The key to this conceptualization is that the experience and reaction of disgust occur earlier, presumably preventing ingestion or contact with a possibly spoiled or unsafe substance [52]. Since disgust is hypothesized to be a primary emotion that developed to avoid disease and contamination [53, 54], individuals with orthorexia may perceive food classified as unhealthy as something that could contaminate and subsequently make them ill. Consequently, this principle could explain orthorexic food avoidance, thus motivating a higher propensity for orthorexia nervosa.

On the other hand, there are many studies in the literature that support the hypothesis that one of the main psychopathological factors in OCD is precisely disgust [7, 55]. Further, a recent meta-analysis showed that ON symptoms are more related to EDs compared to OCD suggesting that ON might belong to the first spectrum. Despite the significant relationships, the non-high magnitude of the associations suggests preliminary evidence that ON is relatively distinct from pre-existing EDs and OCD [56]. Therefore, treating ON as a separate ED seems to be plausible but future studies are needed to better evaluate the association between orthorexia, EDs, and OCD symptoms using longitudinal designs.

Our study has some limitations to take into consideration. Firstly, the tests used are self-report questionnaires, and as such, they can be less precise evaluation tools in investigating the symptomatology since the accuracy and authenticity of the data collected depend on the respondents and their willingness to share the answer. However, online data collection can provide participants with a greater sense of anonymity and privacy, thus obtaining more honest answers. On the other hand, even the online administration of the questionnaires can be considered a limitation due to the lack of any clarifications for the participants. Furthermore, our analyses were conducted on a non-clinical sample, so it would be useful to repeat them using a clinical population, for example with OCD, ON, and EDs. Finally, we found a small positive relationship between ON and disgust sensitivity (*r* = 0.21). According to the widely used cut-off proposed by Cohen [57], our value is between small (0.1) and medium (0.3). However, a more recent and realistic conceptualization of effect sizes in the psychological research deem an *r* = 0.20 as already medium, and the authors actually guard against too big effect sizes (|rs|> 0.4), as they might be too-good-to-be-true [58]. Most importantly, this result provides nomological evidence in favor of the DOS, as it shows a non-zero relationship between levels of ON and levels of pathogen disgust, a finding that was theoretically well-grounded in the literature on the BIS.

Despite these limitations, our study also has several strengths. First, it improves the study by Cerolini and colleagues since it uses a larger sample with a wider age range and, therefore, is more representative of the general population as compared to previous studies on convenience samples of university students. Second, from a psychometric standpoint, we tested the nomological validity of the relationship between orthorexia and disgust sensitivity rather than testing for simple construct validity [33].

## Conclusions

Our results suggested that I-DOS had sound psychometric properties and it can be a useful tool for measuring ON in a general population sample. Further, the most important result was the positive although small relationship between ON and disgust sensitivity. Since disgust has been also associated with other clinical pictures, this preliminary result could be a starting point for future studies that will help to better investigate this phenomenon. Systematically assessing disgust sensitivity could be valuable in clinical practice for specialists and researchers. This assessment would aid in developing targeted therapeutic approaches for individuals who exhibit high levels of disgust sensitivity.

### Supplementary Information

**Additional file 1.** Content of the items of the questionnaires administered.

## Data Availability

The datasets used and analyzed during the current study are available from the corresponding author upon reasonable request.

## Abbreviations

BMI: body mass index
BODS: Body odor disgust scale
BOS: Barcelona orthorexia scale
CFA: Confirmatory factor analysis
CFI: Comparative fit index
DOS: Dusseldorf orthorexia scale
DSM-5-TR: Diagnostic and statistical manual of mental disorders 5-Text revision
EDs: Eating disorders
EHQ: Eating habits questionnaire
ICD-11: International classification of diseases
I-DOS: Italian dusseldorf orthorexia scale
OCD: Obsessive–compulsive disorder
ON: Orthorexia nervosa
ONI: Orthorexia nervosa inventory
RMSEA: Root mean squared error approximation
SRMR: Standardized root mean square residual
TDDS: Three domains of disgust scale
TLI: Tucker-Lewis index
TOS: Teruel orthorexia scale
WLSMV: Weighted least squares—means and variance
