# Supplementary material for: Relationship between disgust and orthorexia nervosa and psychometric properties of the Italian Dusseldorf orthorexia scale in a general population sample
Source: J Eat Disord. 2023 Oct 3;11:174. doi: 10.1186/s40337-023-00899-5 (PMC10546774; doi:10.1186/s40337-023-00899-5)
Supplement: Supplementary file 1 — Additional file 1. Content of the items of the questionnaires administered. [file 40337_2023_899_MOESM1_ESM.docx]

**Supplementary file 1. Content of the items of the questionnaires administered.**

**Düsseldorf Orthorexie Scale (DOS, Chard et al., 2018)**

| 1. Eating healthy food is more important to me than indulgence/enjoying the food. |
| --- |
| 1. I have certain nutrition rules that I adhere to. |
| 1. I can only enjoy eating foods considered healthy. |
| 1. I try to avoid getting invited over to friends for dinner if I know that they do not pay attention to healthy nutrition. |
| 1. I like that I pay more attention to healthy nutrition than other people. |
| 1. If I eat something I consider unhealthy, I feel really bad. |
| 1. I have the feeling of being excluded by my friends and colleagues due to my strict nutrition rules. |
| 1. My thoughts constantly revolve around healthy nutrition and I organize my day around it. |
| 1. I find it difficult to go against my personal dietary rules. |
| 1. I feel upset after eating unhealthy foods. |

*Notes: 5-point Likert scale, ranging from 1 (this does not apply to me) to 4 (this applies to me).*

**Body Odors Disgust Scale (BODS, Liuzza et al., 2017)**

| 1. You are alone at home and notice that the T-shirt you are wearing smells strongly from your own sweat. |
| --- |
| 1. You are standing next to a stranger and notice that the T-shirt they are wearing smells strongly from their sweat. |
| 1. You are alone at home and notice that your feet smell strongly. |
| 1. You are sitting next to a stranger and notice that their feet smell strongly. |
| 1. You are alone at home and notice that your breath smells strongly. |
| 1. You are chatting with a stranger and notice that their breath smells strongly. |
| 1. While alone at home, you use the bathroom. Afterward, you notice that the room smells strongly of your feces. |
| 1. You use the bathroom after a stranger and notice that the room smells strongly of their feces. |
| 1. You are alone at home and pass gas. It is silent but smells strongly. |
| 1. You are sitting next to a stranger and they pass gas. It is silent but smells strongly. |
| 1. While alone at home, you use the bathroom. Afterward, you notice that the room smells strongly of your urine. |
| 1. You use the bathroom after a stranger and notice that the room smells strongly of their urine. |

*Notess: five-point Likert scale, ranging from 1 (not at all disgusting) to 5 (extremely disgusting).*

**Three Domains of Disgust Scale– Pathogen domain (TDDS, Tybur et al., 2009)**

| 1. Stepping on dog poop. |
| --- |
| 1. Sitting next to someone who has red sores on their arm. |
| 1. Shaking hands with a stranger who has sweaty palms. |
| 1. Seeing some mold on old leftovers in your refrigerator. |
| 1. Standing close to a person who has body odor. |
| 1. Seeing a cockroach run across the floor. |
| 1. Accidentally touching a person’s bloody cut. |

*Notes: seven-point Likert scale, ranging from 0 (not at all disgusting) to 7 (extremely disgusting).*
